# Photocatalytic Semi-Hydrogenation of Alkynes: A Game of Kinetics, Selectivity and Critical Timing

**DOI:** 10.3390/nano13172390

**Published:** 2023-08-22

**Authors:** Melissa Cely-Pinto, Bowen Wang, Juan C. Scaiano

**Affiliations:** Department of Chemistry and Biomolecular Sciences, University of Ottawa, Ottawa, ON K1N 6N5, Canada; mcely097@uottawa.ca (M.C.-P.); bowenwanguv@gmail.com (B.W.)

**Keywords:** photochemistry, heterogeneous catalysis, semi-hydrogenation, supported catalysts

## Abstract

The semi-hydrogenation reaction of alkynes is important in the fine chemicals and pharmaceutical industries, and it is thus important to find catalytic processes that will drive the reaction efficiently and at a low cost. The real challenge is to drive the alkyne-to-alkene reaction while avoiding over-hydrogenation to the saturated alkane moiety. The problem is more difficult when dealing with aromatic substitution at the alkyne center. Simple photocatalysts based on Palladium tend to proceed to the alkane, and stopping at the alkene with good selectivity requires very precise timing with basically no timing tolerance. We report here that the goal of high conversion with high selectivity could be achieved with TiO_2_-supported copper (Cu@TiO_2_), although with slower kinetics than for Pd@TiO_2_. A novel bimetallic catalyst, namely, CuPd@TiO_2_ (0.8% Cu and 0.05% Pd), with methanol as the hydrogen source could improve the kinetics by 50% with respect to Cu@TiO_2_, while achieving selectivities over 95% and with exceptional timing tolerance. Further, the low Palladium content minimizes its use, as Palladium is regarded as an element at risk of depletion.

## 1. Introduction

The semi-hydrogenation of alkynes to alkenes is one of the most important reactions in several industrial processes. Among the different hydrogenation reactions, the semi-hydrogenation of alkynes to alkenes is particularly challenging and important to the manufacture of bulk and fine chemicals. These reactions are commonly performed over supported Pd catalysts, which can be modified by the addition of different metals, including silver [1], or even using CO, which can act as a reversible poison [2,3]. For example, reports show that the semi-hydrogenation of phenylacetylene was performed using Pd nanoparticles in the forms of dispersed colloids [4] and supported systems [5,6]. The latter is more commercially attractive for industrial applications given their better handling properties, including post-reaction separation, and therefore, the development of heterogeneous catalysts with interesting compositions, high selectivity and catalytic efficiency will contribute to sustainable chemistry [7]. Different approaches have been explored, such as the one disclosed by Soberanas et al. [8], who synthesized solid-supported Pd-(CaCO_3_)_n_ clusters, showing a high catalytic activity for the semi-hydrogenation of internal alkynes compared with terminal alkynes, wherein, for example, internal aliphatic alkynes reacted faster with the catalyst than the aromatic ones. On the other hand, Wang et al. [9] proposed Pd-Se nanocrystals to enable the semi-hydrogenation of phenylacetylene to styrene, demonstrating the σ-π associative adsorption of the C≡C bond but suppressing the hydrogenation of C=C bond, which results in high selectivity. Zheng et al. [10] also worked with Pd, but in their case, they demonstrated that alloyed Pd-Pb on precipitated calcium carbonate (PCC) supports could be an upgraded version of the Lindlar catalyst that provides promising results for the semi-hydrogenation of phenylacetylene to styrene. Further, Lv et al. [11] developed a novel alumina-supported Pd catalyst for the selective hydrogenation of phenylacetylene and less production of the byproduct ethylbenzene given the good dispersion of Pd and strong metal-support interaction capable of creating more active sites for hydrogenation and facilitating the desorption of the alkene.

However, even though different investigations have focused on increasing the selectivity of alkenes using different materials, the conversion of alkynes to alkanes is a straightforward process and can be performed quite efficiently if moieties other than the target alkyne are not affected in the process. Therefore, heterogeneous photocatalysts based on palladium over TiO_2_ could be an alternative to the disclosed ones since they show excellent performance and the control over the light source might help during these processes. Different alternatives directed toward depositing metals over TiO_2_ have also been explored as an effective approach to different materials in semi-hydrogenation reactions given its photon harvesting capacity and the increased charge separation that helps improve the semiconductor photocatalyst activity [12]. For example, such is the case for TiO_2_ decorated with palladium nanoparticles (Pd@TiO_2_). Some catalysts, like Pd@TiO_2_, are excellent, and while the rate of first step k_1_ tends to be faster than k_2_, in reality, it is very difficult to stop at the critical point in time to achieve high selectivity. This is conceptually illustrated in Figure S1, where even if k_1_ is 10 times faster than k_2_ (panel B), only about 70% selectivity and about 70% alkene yield are obtained at 99% conversion. In contrast, when k_2_ is 100 times slower than k_1_ (panel C), the selectivity and yield are about 97% at 99% conversion. In other words, an alkene-selective catalyst must show very slow hydrogenation of alkenes while having an acceptable rate constant for alkyne hydrogenation.

Figure S1 is based on a very simple kinetic simulation, where the total time is equal to five lifetimes for the reagent decay according to first-order kinetics, reducing its concentration approximately 150 times. Heterogenous catalysis may not follow a simple kinetic model, but in any event, Figure S1 serves to illustrate the point. The expression “critical timing” used in the title relates to cases where it is possible to achieve reasonable selectivity in very fast reactions only if the reaction is stopped at a very precise time, while letting the reaction go for a bit longer (e.g., 20% longer) totally destroys the selectivity. The reality of the organic chemistry laboratory is that reaction times must be somewhat forgiving, as reaction times are measured in hours or overnight and rarely on continuous real-time monitoring of the reaction mixture composition.

**Scheme 1:**
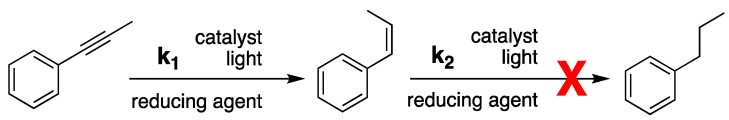
Reaction path for semi-hydrogenation of 1-phenyl-1-propyne (PhP) using a photocatalyst. In an ideal situation k_2_ << k_1_.

Based on the above, semi-hydrogenation of alkynes can be a challenging process, as one would like to combine reasonable activity with high selectivity, such as illustrated in Scheme 1 for the case of 1-phenyl-1-propyne.

On the other hand, other than Pd, different metals have been used in these reactions, for example, Cu on TiO_2_ (Cu@TiO_2_) showed sharp chemoselectivity when hydrogenating internal alkynes to alkenes without being further hydrogenated to alkanes [13]. However, according to Imai et al., when performing photocatalytic semi-hydrogenation of alkynes with Cu-Pd on TiO_2_ (CuPd@TiO_2_), the reaction works very well with aliphatic systems, but, for example, for 1-phenyl-acetylene in 2-propanol, the selectivity is reduced to 66% due to ongoing hydrogenation to ethylbenzene [14].

In this sense, the time of the reaction might be improved using a combination of two different elements. Additionally, beyond the hydrogenation kinetics on the surface of the catalyst, it is important to consider that selectivity is also controlled by the adsorption strength and the configuration of the reactants and intermediates on the surface of catalysts [15], and thus, these are parameters to consider when performing these reactions. Consequently, the ideal catalyst should prevent the over-hydrogenation of the formed alkene while keeping acceptable reaction rates, selectivity and reactivity of the catalysts in these reactions.

Here, we compared catalysts based on Pd or Cu on TiO_2_ (Pd@TiO_2_ and Cu@TiO_2_), as well as the mixture of both metals on TiO_2_ (CuPd@TiO_2_), with the last one prepared via galvanic exchange. We examined several alkynes, but frequently concentrated our detailed studies on 1-phenyl-1-propyne. All measurements included a detailed time profile showing the evolution of the reaction, avoiding end-point studies where conversions and selectivity measurements can reflect a poor choice of reaction times or irradiance values. The solvents and hydrogen donors were ethanol (EtOH) and methanol (MeOH) and the reactions were driven using UVA irradiation, which is a spectral region largely absorbed by TiO_2_.

## 2. Results and Discussion

We tested three different catalysts and several alkynes. For selected combinations, we also examined the adsorption of the alkynes on the different materials, something that earlier work showed to be very important [15]. Finally, we looked at the catalytic properties of the materials, and in all cases, we examined the time dependence in detail. As Figure S1 illustrates, both the conversion and selectivity were critically dependent on time, and thus, end-point analysis was not particularly informative.

### 2.1. Catalyst Synthesis

Pd@TiO_2_: Based on a reported synthesis [16,17,18]. Approximately 15 mg of metal precursor salt (PdCl_2_) was mixed with TiO_2_ (~500 mg) in 150 mL of Milli-Q water and sonicated for 20 min. The slurry was irradiated in a Luzchem photoreactor equipped with 14 UVA bulbs for 24 h with vigorous stirring in a crystallizing Pyrex^®^ dish with a Pyrex^®^ cover. The slurry was centrifuged and washed with Milli-Q water four times to remove unreacted metal precursor salt and dried overnight in a desiccator under vacuum. The obtained powder had a dark grey color.

Cu@TiO_2_: Based on a reported synthesis [19]. A total of 33 mg of CuCl_2_·2H_2_O were mixed with TiO_2_ (~500 mg) and 80 mg of Irgacure-2959 in 130 mL of Milli-Q water and purged with argon for 15 min. After this, the slurry was irradiated in a Luzchem photoreactor equipped with 14 UVA bulbs for 24 h with vigorous stirring. The slurry was filtered and washed with Milli-Q water at least seven times to remove unreacted metal precursor salt and dried overnight in a desiccator under vacuum. The powder had an intense beige color with green tones.

CuPd@TiO_2_: The catalyst was obtained via galvanic replacement synthesis, which relies on the spontaneous redox reaction between the atoms of a substrate (Cu@TiO_2_ previously synthesized) and the salt precursor (Pd(acac)_2_ in this case) of another metal. Specifically, 0.28 mg of Pd(acac)_2_ was mixed with 20 mL of Milli-Q water and sonicated for 15 min. After completing this, 10 mL of the solution was taken and mixed with 50 mg of Cu@TiO_2_. The slurry was purged with argon for 15 min and vigorously stirred overnight. The slurry was filtered and washed with Milli-Q water at least seven times and dried overnight in a desiccator under vacuum. The powder had a similar color to the one from Cu@TiO_2_.

### 2.2. Characterization

**Figure 1:**
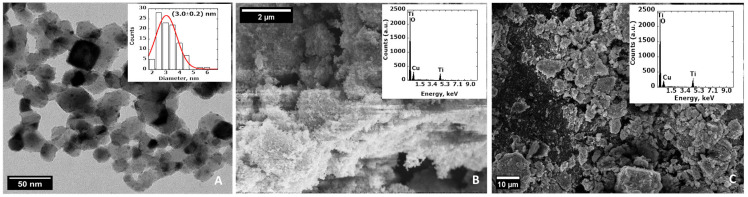
(**A**) TEM image of Pd@TiO_2_ nanoparticles (histogram made with 100 particles (size bar is 50 nm). The mean particle size was 3.0 ± 0.2 nm. (**B**) SEM and EDS results for Cu@TiO_2_. EDS results show the peaks for Ti, O and Cu, confirming the presence of Cu in the catalyst. (**C**) SEM and EDS results for CuPd@TiO_2_. EDS results show the peaks for Ti, O and Cu, which corroborate the presence of Cu in the catalyst. However, it was not possible to identify the peak for Pd since the loading of this metal was too low (0.05% according to ICP).

PdNP morphologies were approximately spherical. Transmission electron microscopy (TEM) images and a histogram for PdNPs are displayed in Figure 1. Additionally, for Cu@TiO_2_ and CuPd@TiO_2_, scanning electron microscopy (SEM) and energy-dispersive X-ray spectroscopy (EDS) were used to determine the presence of Cu and Pd in the catalysts. The TEM image of Pd@TiO_2_ nanoparticles in panel A of Figure 1 shows a mean particle size of 3.0 ± 0.2 nm. On the other hand, according to EDS results of panels B and C of Figure 1, for Cu@TiO_2_ and CuPd@TiO_2_, the presence of Cu was identified for both catalysts, but it was not possible to identify the peak of Pd in CuPd@TiO_2_ since the metal loading was too little (Table 1). X-ray photoelectron spectroscopy (XPS) analyses of Pd@TiO_2_ and Cu@TiO_2_ were conducted and published in previous publications, suggesting the presence of PdO with a small contribution of more reduced palladium species for Pd@TiO_2_ [18] and satellite peaks of Cu (II) [20].

The metal loadings were determined using inductively coupled plasma optical emission spectroscopy (ICP-OES) analysis and are disclosed in Table 1.

### 2.3. Alkyne Adsorption on Selected Materials

**Figure 2:**
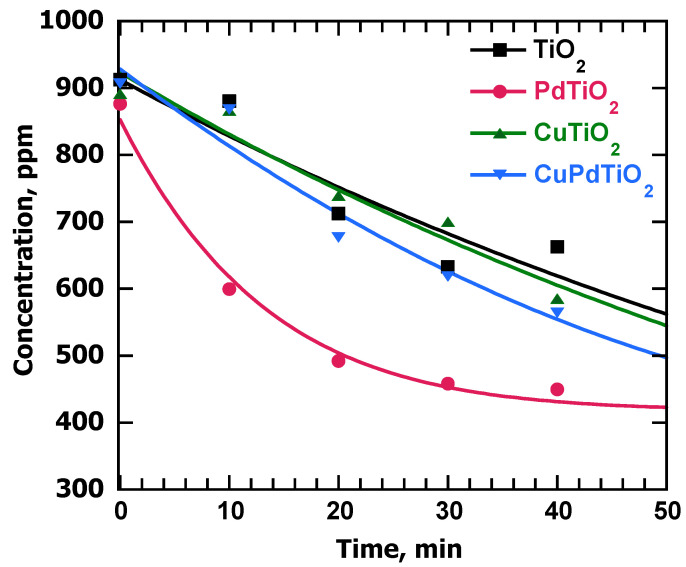
Adsorption experiments of PhA using the synthesized catalysts. Conditions: 10 mg catalyst; 1 mL of 900 ppm alkyne solution in ethanol. Stirring times: 0 min, 10 min, 20 min, 30 min and 40 min. Aliquots were taken at these times and filtered before using GC-MS to monitor the area under the peak of PhA at different times (3.70 min retention time identified using gas chromatography (GC), as shown in Figure S2, while Figure S3 shows the mass spectra of the alkynes)**.** Calibration curves for every alkyne were necessary to determine the concentrations of the alkyne at the mentioned times using 3,5-Di-tert-butyltoluene as an external standard.

Alkyne adsorption experiments are essential when looking at catalyzed reactions given that they might occur at the catalyst surface. In the present work, adsorption experiments were performed using two representative alkynes: 900 ppm solutions of phenylacetylene (PhA) and 1-phenyl-1-propyne (PhP) were stirred for 40 min in the presence of 10 mg of each material in EtOH. Preliminary experiments showed that 40 min was an adequate time to reach a plateau, that is, adsorption equilibration. A representative plot shown in Figure 2 and Table 2 summarizes the relevant data. Figure S4 also shows the results for PhP.

Examination of Table 2 shows that the metals played a key role in enhancing the adsorption properties of the TiO_2_-supported systems. Note, for example, that in the case of PhP, 2% palladium content was enough to triple the amount of alkyne adsorbed when compared with TiO_2_ itself. These data show that there was a significant number of sites over the Pd catalyst that strongly adsorbed the alkyne molecules. On the other hand, the effect of copper was also clear, but not as large as that of palladium. Additionally, the bimetallic catalyst exhibited similar adsorption to the one shown by Cu@TiO_2_, even if the alkyne was internal. Also important, when preparing samples for catalysis studies, one must wait at least 30 min to ensure that the adsorption equilibrium is reached. Unfortunately, this information is rarely included in literature reports, but it is essential to understand different types of reactions, like semi-hydrogenation ones.

### 2.4. Semi-Hydrogenation of Alkynes with Single-Metal TiO_2_ Catalysts

**Scheme 2:**
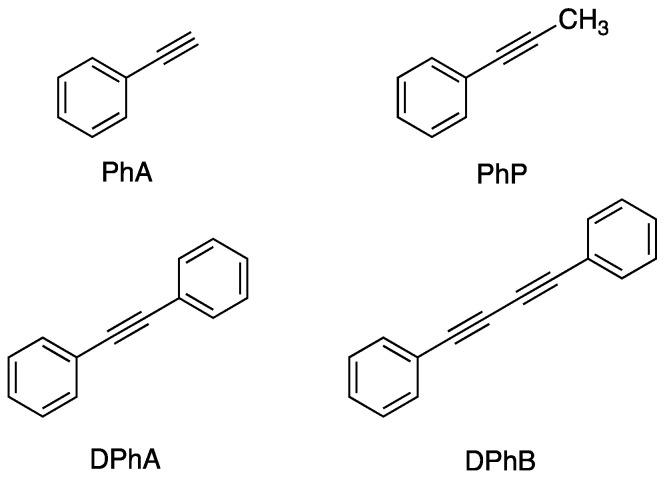
Alkynes studied in this research.

Scheme 2 shows the alkynes examined in this work. Among these, PhA and PhP were examined in more detail.

**Figure 3:**
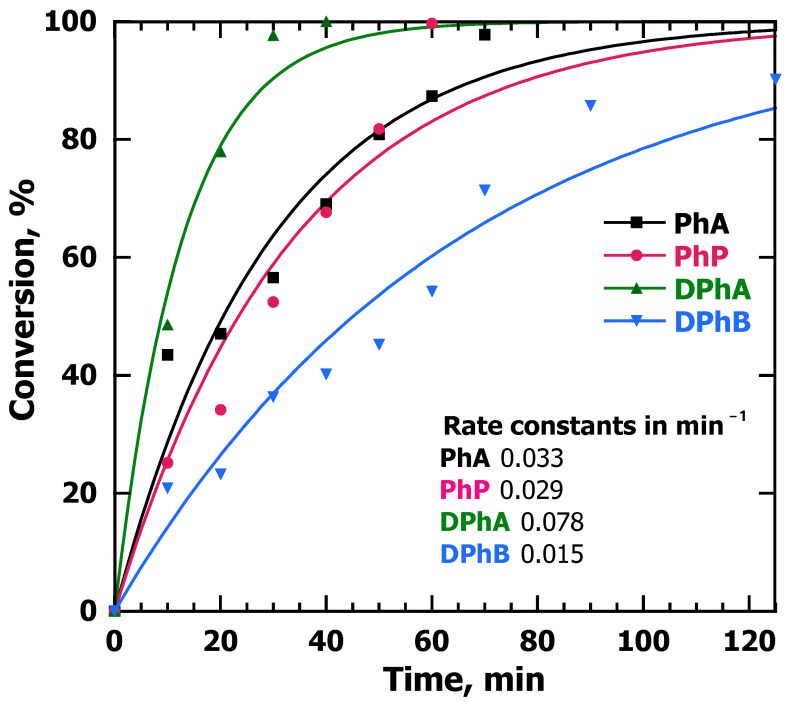
Conversion of alkynes after performing hydrogenation reactions using alkyne solutions in EtOH at a concentration of 6 mM under argon and irradiated with UVA light at 25% power and with 10 mg Pd@TiO_2_ suspended in 8 mL. Under these conditions, the order of relative consumption rates changes, with DPhA showing the fastest consumption, while DPhB was the slowest one. Note that before these experiments, semi-hydrogenation reactions using 900 ppm as the main concentration for the alkynes were also performed. In this case, the molar concentrations were not the same for all alkynes (Figure S7), but rather the mass concentration was matched.

All our studies involved irradiation with an LED UVA source centered at 370 nm (FWHM ~30 nm) with an adjustable irradiance (Figure S5). Under typical exposure conditions (4 cm from the emitter), the irradiance at 100% power was ~320 W/m^2^, although when using Pd@TiO_2_, most studies were performed at 25% power (~87 W/m^2^) (Figure S6). Figure 3 shows the conversion against time for the four alkynes at 6 mM studied when the catalyst was Pd@TiO_2_ and the hydrogen donor was EtOH.

**Figure 4:**
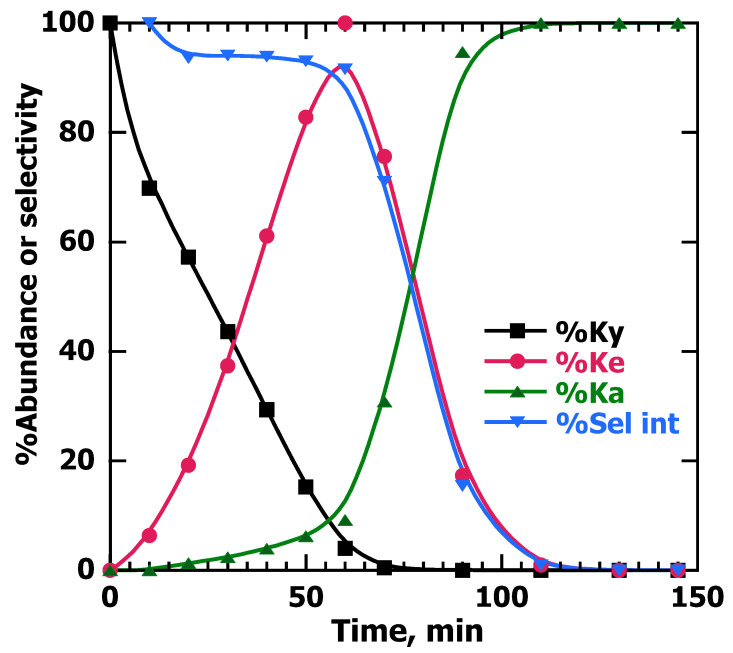
Abundance of the PhP (%Ky), along with alkenes (%Ke) and alkane (%Ka), and selectivity toward the alkenes (%Sel int) vs. time for 8 mL of a 6 mM solution in EtOH of PhP stirred with 10 mg of Pd@TiO_2_. The reaction was performed in a Pyrex test tube with a rubber cap and irradiated with 25% UVA power under Ar. At 60 min, the abundance of the alkyne was more than 95% and the selectivity toward alkenes was 91%. Only 10 min longer made the selectivity 70% at 70 min.

It was interesting to examine the performance of the alkynes with Pd@TiO_2_ and EtOH as a solvent as a function of time. This is illustrated for PhP in Figure 4. The reaction was quite fast, reaching completion in under 80 min and a maximum yield of predominantly *cis* alkene at about 60 min when the remaining PhP was 4% and the selectivity toward alkenes was more than 90%, with about ~10% of 1-phenylpropane already formed at this point. Concerning the isomers formed in these reactions, in Figure S8 there is evidence that traces of the *trans*-isomer (retention time of 5.22 min in the GC chromatogram) were also formed, but the major product was the *cis*-compound (retention time of 4.90 min). Mechanistically, the reaction involves strong coordination between H (from the H source, in this case, the solvent) with Pd atoms in crystal lattices, and thus, the population of Pd sites was proposed to govern the hydrogenation events on the metal surface [21].

Continuing with the analysis of Figure 4, it is worth highlighting that at times slightly longer than that required for alkyne consumption, the selectivity was reduced to 70% at 70 min and an additional 20 min reduced it to less than 20% (see “%Sel int” in Figure 4). This brings in the concept of critical timing, where in this case, it is not possible to achieve reasonable selectivity given that the reaction is very fast, and thus, stopping it at a very precise time might be difficult when working with Pd@TiO_2_. It is possible to use Palladium catalysts for semi-hydrogenation, but the time at which the reaction must be stopped requires critical timing and an error of just a few minutes induces extensive over-hydrogenation to the alkane product.

**Figure 5:**
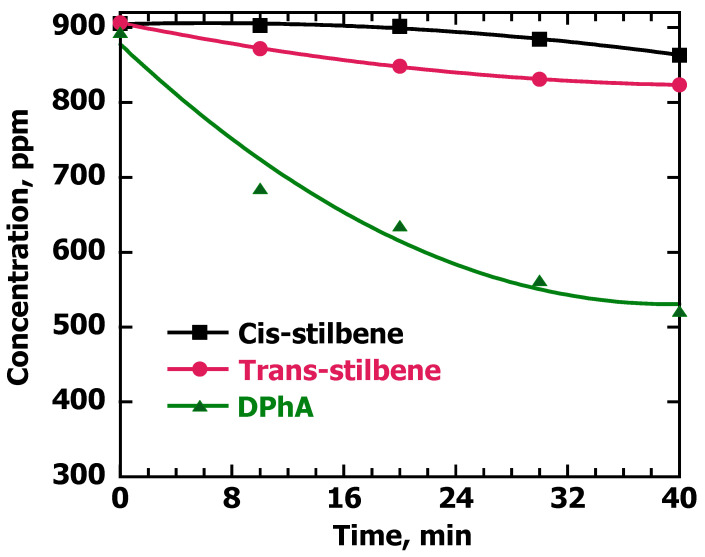
Adsorption of DPhA, cis and trans-stilbene on Pd@TiO_2_. Solutions of the compounds in EtOH (900 ppm) and 10 mg of Pd@TiO_2_ were stirred for 0, 10, 20, 30 and 40 min. Aliquots of the solution were taken at these times and filtered before analyzing them via GC-MS. The chromatograms provided via GC-MS were analyzed using calibration curves to determine the concentration of the compounds, where 3,5-di-tert-butyltoluene was used as an external standard.

The problem of critical timing occurs in spite of the fact that alkenes show less affinity for adsorption on the Pd@TiO_2_ catalyst than alkynes. To verify this hypothesis, we tested the adsorption of DPhA vs. *cis* and *trans*-stilbene on Pd@TiO_2_. Figure 5 shows these results obtained after stirring 1 mL of a 900 ppm solution for DPhA with 10 mg of Pd@TiO_2_ for 40 min. A total of 41% adsorption was achieved since at 40 min, the concentration of the alkyne was reduced from 900 ppm to 520 ppm. Moreover, for the *cis* and *trans* isomers of stilbene, after 40 min, the adsorption on the surface catalyst was only 5% and 9%, respectively. Consequently, the adsorption of the alkyne on the catalyst surface was more efficient compared with the alkenes. For the semi-hydrogenation of alkynes, the literature suggests that alkynes adsorb more selectively on a metal surface, retarding alkene adsorption and hindering its hydrogenation to an alkane, as long as alkynes are still present in the reaction mixture [22]; these results might help to understand the semi-hydrogenation process.

The issue of critical timing illustrated in Figure 4 for PhP is not unique and similar problems are found for the other alkynes studied in this work (Figure S9) and summarized in Table 3. The loss of selectivity is rapid and large, which makes stopping the reaction at a critical time essential. Even 15 min will make the loss of selectivity quite large. For PhP, illustrated in Figure 4, a difference of 15 min with respect to t_95_ will reduce the selectivity to 59%, while stopping 15 min too soon will reduce the conversion and yield to 78%.

We also examined the effect of catalyst loading, illustrated in Figure S10 for PhP. We note that a reduction by a factor of five in the catalyst loading (2 mg rather than 10 mg of Pd@TiO_2_) only reduced the rate of consumption by a factor of two. However, the issue of critical timing discussed in relation to Figure 4 and Table 3 remains unchanged. In general, small deviations from the critical time can cause large reductions in selectivity. Further, stopping early is not a solution either because the conversion curve is very steep and early stops lead to reduced conversion and yields.

**Figure 6:**
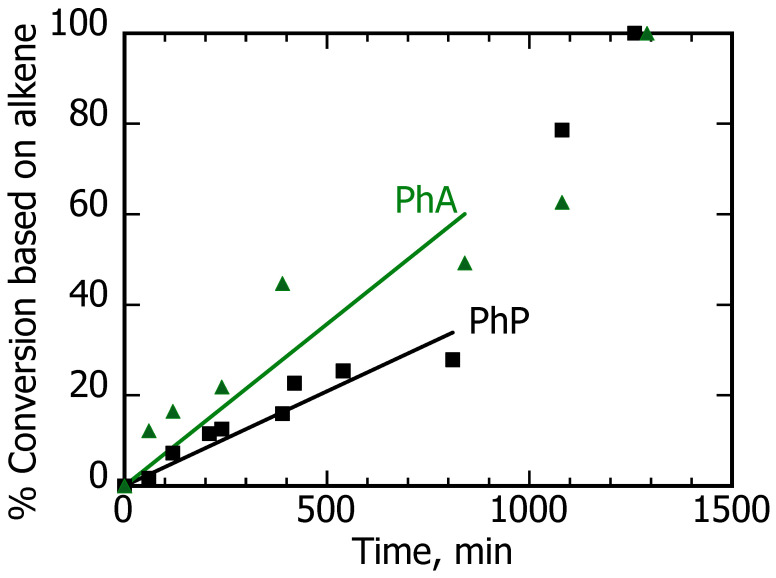
Conversion vs. time for PhA (green) and PhP (black). The samples consisted of 8 mL of 6 mM solutions in EtOH of the alkynes stirred with 10 mg of Cu@TiO_2_ in different Pyrex tubes and irradiated with 25% UVA power under Ar. The fitting corresponds to low conversion (<50%) data and shows that PhA was 70% faster than PhP. The cis-alkene was essentially the only product with a selectivity exceeding 98% for the first 1000 min.

The situation with Cu@TiO_2_ as a catalyst was very different from that with Pd@TiO_2_. The paragraphs and figures that follow elaborate on these differences. In brief, the copper catalyst was slower than palladium, but in terms of critical timing, it was very forgiving, and over-hydrogenation was not a problem. In the qualitative context of Figure 1, one would estimate that k_2_ < 0.001 k_1_. Initial experiments in this section were performed with 6 mM PhP and PhA in EtOH using 100% lamp power since when using 25%, the reaction can take days. Figure 6 shows the time evolution of the reaction over an extended period and with 100% lamp power. Even doubling the time required for 100% conversion, the yield of alkanes was minimal.

For Cu@TiO_2_ in EtOH, there was no formation of 1-phenylpropane, even at longer times in comparison with the results obtained with Pd@TiO_2_ (compare with Table 3). Copper showed a major improvement in the selectivity of the alkynes, even if the reaction was extended for 20 h. Accordingly, over-hydrogenation was not a problem, yet, the reaction time was around 10 times longer than that for Pd@TiO_2_. Thus, the issue of longer reaction rates needs improvement.

**Figure 7:**
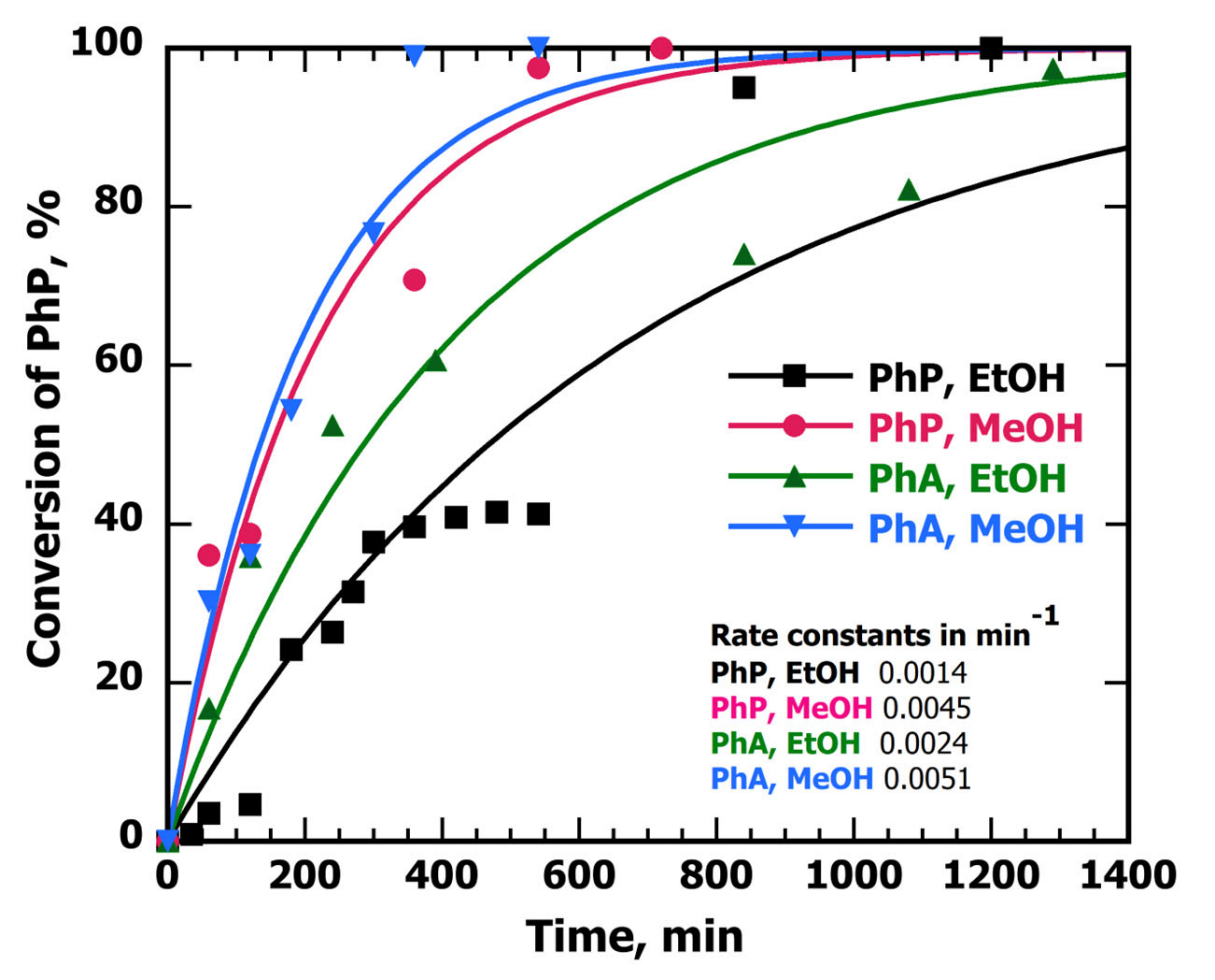
Conversion of PhA and PhP in MeOH and EtOH vs. time. A total of 8 mL of 6 mM solutions in MeOH and EtOH of the alkynes were stirred with 10 mg of Cu@TiO_2_ in different Pyrex^®^ containers and irradiated with 100% UVA power under Ar. The data were fit with an exponential forced fit to start at zero and level off at 100%; the curves are mostly presented to help the reader visualize data trends.

In an attempt to improve the kinetics, we tried using a different solvent that may help to maintain the selectivity toward the alkenes and reduce reaction times [23]. According to this, MeOH was used as the hydrogen donor/solvent given its promising results in catalytic hydrogenation reactions. Hence, keeping the same conditions of the semi-hydrogenation reactions, Figure 7 shows the time profiles for PhP and PhA when performing the semi-hydrogenation reactions in EtOH and MeOH.

The results of Figure 7 show that MeOH, as both the hydrogen donor and solvent, increased the rates of reaction. These results show that the rate of alkyne-to-alkene reaction when using Cu@TiO_2_ and MeOH could be improved while avoiding over-hydrogenation to the saturated alkane moiety. Control experiments were also performed and Figure S12 shows the results for the reaction of PhP in MeOH with TiO_2_ itself, where after 540 min (9 h) of reaction, less than 5% of PhP was consumed and barely around ~1% of *cis*-1-phenyl-1-propene was produced. This confirms that decorating TiO_2_ with a metal improved the photocatalytic activity of the material for these reactions.

### 2.5. Semi-Hydrogenation of Alkynes with the Bimetallic CuPd@TiO_2_ Catalyst

**Figure 8:**
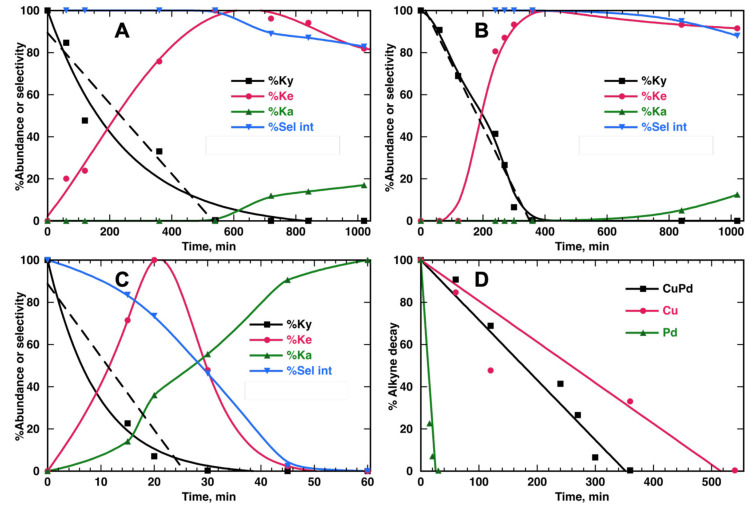
(**A**) Comparison of catalytic performance for 8 mL of 6 mM PhP solution in MeOH stirred with 10 mg of catalyst and irradiated with 25% UVA power under Ar for Cu@TiO_2_, (**B**) CuPd@TiO_2_ and (**C**) Pd@TiO_2_. Panel (**D**) compares the different consumption slopes (100% to 5%) for the alkyne with the different catalysts. The kinetic data are also summarized in Table 4. Dashed lines fit the early linear data.

The motivation to test a bimetallic catalyst relates to the excellent selectivity of Cu@TiO_2_ and the excellent kinetics of Pd@TiO_2_. Based on previous results, we wondered whether light-initiated incorporation of palladium to the Cu@TiO_2_ catalyst may help with the kinetics without significant loss of selectivity and without creating an important critical timing issue. With this in mind, we prepared CuPd@TiO_2_ via galvanic replacement, with a loading of 0.8% copper and 0.05% palladium. The catalyst was tested in a MeOH suspension with solutions of 6 mM PhP in MeOH and two different catalyst loadings, namely, 10 mg and 5.4 mg, which are shown in Figure S11. The choice of MeOH reflects the observation of improved kinetics shown in Figure 7. In order to compare the different catalysts, we selected 6 mM of PhP and 10 mg of the catalyst under the conditions specified in the caption of Figure 8. Please note that the CuPd@TiO_2_ catalyst was prepared from a sample of the same Cu@TiO_2_ material used in the experiment.

Table 4 shows that incorporating traces of Pd (see Table 1) was enough to increase the rate by 52% with respect to Cu@TiO_2_, and it even remained much slower than Pd@TiO_2_.

The observed performance of the bimetallic CuPd@TiO_2_ catalyst was improved over previously reported catalysts, including the Lindlar Pd catalyst and other systems where, for example, the addition of organic ligands is needed to avoid over-hydrogenation of alkenes to alkane [24,25]. Along this line of thought and in agreement with previous reports based on Pd bimetallic catalysts [26,27], the possible absence of substantial Pd agglomeration results in weak adsorption toward alkene on the catalyst surface and high alkene selectivity. It is also remarkable that panel B in Figure 8 shows better long-term selectivity than panel A. Surprisingly, a small amount of Pd not only increased the rate of reaction but caused a selectivity improvement. Additionally, it is worth highlighting that the light source was essential for photocatalytic reactions like these. According to the results of Figure S13, when the reaction for PhP and 10 mg of CuPd@TiO_2_ was performed without light, for at least 9 h, there was no consumption of the alkyne nor production of alkenes.

**Figure 9:**
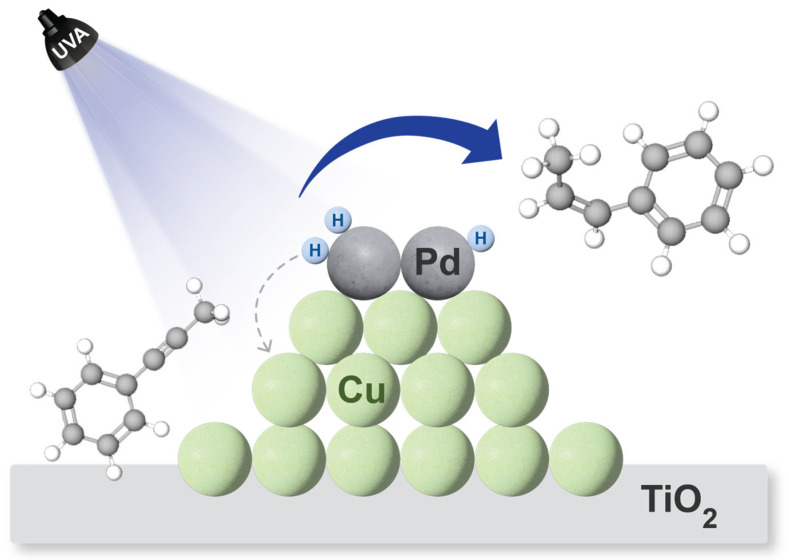
Plausible scenario to understand the activity shown by CuPd@TiO_2_. Figure inspired by similar concepts in the literature [28].

The mechanism by which palladium improves reactivity without a decrease in selectivity is probably similar to that for palladium on gold, where it is believed that hydrogen atoms generated on Pd leak into the second metal where the reaction takes place [28]. In this case, H atoms from the solvent, either EtOH or MeOH, generated on Pd would be transferred to the copper that controls the selectivity of the reaction, as depicted in Figure 9.

Therefore, introducing Pd, which can rapidly speed up the reaction, on the Cu/TiO_2_ catalyst, which has been shown to be much more chemoselective, helped to improve the selectivity toward alkenes in this photocatalytic reaction and the critical timing control was forgiving when reaction times were measured in hours or overnight.

## 3. Materials and Methods

### 3.1. Materials

Chemicals and materials were sourced from multiple suppliers for the experimental work. The initiator Irgacure-2959 was a gift from BASF, while the alkynes, solvents (spectrophotometric grade >99.8%), PdCl_2_, CuCl_2_·2H_2_O and Pd(acac)_2_ were purchased from Sigma Aldrich. TiO_2_ P25 was purchased from Univar Canada. The MilliQ water was obtained via purification of deionized water using a Thermo Scientific^TM^ Barnstead^TM^ GenPure^TM^ (Waltham, MA, USA)water purification system (conductivity 18 MΩ/cm).

### 3.2. Instruments

The photocatalytic performance of the catalysts at the bench scale was examined in Pyrex^®^ test tubes. The reaction mixture was stirred and irradiated using a UVA Kessil lamp PR160-370 nm with a controllable LED at different powers (25% and 100%). The synthesis of the catalysts was performed by using a Luzchem photoreactor equipped with 14 UVA bulbs. Transmission electron microscope (TEM) images were acquired at the University of Ottawa’s Materials Characterization Facility (MatChar) with a JEM2100F FETEM (JEOL, Tokyo, Japan) field emission transmission electron microscope. Particle sizes were determined with ImageJ analysis. Scanning electron microscopy (SEM, Zeiss, Jena, Germany) and energy-dispersive X-ray spectroscopy (EDS, Bruker, Milton, ON, Canada) images were acquired at the NanoFab Facility at the University of Ottawa with a Zeiss GeminiSEM 500 column using a Schottky thermal field emitter filament for imaging between 0.1 and 30 kV for sub-nanometer resolution imaging and with a Bruker EDS detector suitable for X-ray detection for spot identification of the composite. The metal loading of catalysts was determined using inductively coupled plasma emission spectrometry (ICP-OES) using an Agilent Vista Pro ICP Emission Spectrometer (Santa Clara, CA, USA). Characterization of the semi-hydrogenation reactions and their products was performed by using mass spectrometry in an Agilent 6890-N Gas Chromatograph with an Agilent 5973 mass selective detector (Santa Clara, CA, USA).

### 3.3. Semi-Hydrogenation Reactions

The reactions were performed in 15 mL Pyrex^®^ test tubes with a rubber cap. In a typical reaction, 8 mL of 900 ppm or 6 mM alkyne solution in EtOH was stirred with 10 mg of catalyst (Pd@TiO_2_, Cu@TiO_2_) and irradiated with a 25% or 100% UVA light source at 4 cm from the test tube. The reaction mixture was purged with argon. Similar preparations were also made using methanol as the solvent and using either the mentioned catalysts or CuPd@TiO_2_. Aliquots were taken at regular intervals and filtered using syringe filters with a PTFE membrane in order to remove the catalyst before using an Agilent 6890-N Gas Chromatograph with an Agilent 5973 mass selective detector to monitor the reactions and identify the peaks of the reactants and products. Every time the aliquots were taken, purging the reaction with Ar was necessary in order to assure the inert environment. Calibration curves for every alkyne were necessary to determine the concentration of the alkyne at the mentioned times using 3,5-di-tert-butyltoluene as an external standard.

## 4. Conclusions

Semi-hydrogenations always present significant challenges, as the interplay of kinetics and selectivity determine the performance of the catalyst. The reality of the organic laboratory is that reactions are not stopped at exact times and reaction end points are frequently controlled by convenience, such as in the case of “overnight reactions”. Reactions that can lose their yield or selectivity over short periods are simply impractical. Such is the case of Pd@TiO_2_ as a catalyst, where critical timing is essential to preserve high yields and selectivity. In contrast, Cu@TiO_2_ and CuPd@TiO_2_ offer excellent selectivity without critical timing requirements. The latter was about 52% faster than Cu@TiO_2_ and to our surprise, not only preserved the selectivity but also offered a small improvement (compare Figure 9 panels A and B). Thus, for alkyne semi-hydrogenation, we recommend CuPd@TiO_2_ as a catalyst and methanol as the solvent. These results open the way to synthesizing highly active and selective catalysts inspired by the reality of timing control of semi-hydrogenation processes at the bench scale. However, future work should focus on further exploration to optimize even more the time of the reaction to complete the semi-hydrogenation process in less time but without losing great selectivity and still having control over the critical point between progressing from an alkene to an alkane.

## Figures and Tables

**Table 1:** ICP-OES analysis of the catalysts before performing the adsorption experiments and semi-hydrogenation reactions.

Material	Metal Loading (%)
Pd@TiO_2_	2.0 wt % Pd
Cu@TiO_2_	0.9 wt % Cu
CuPd@TiO_2_	0.8 wt % Cu and 0.05 wt % Pd

**Table 2:** Adsorption capacity of the catalysts when being in contact with PhA and PhP after 40 min of stirring.

Material	Phenylacetylene (PhA)	1-Phenyl-1-Propyne (PhP)
TiO_2_	27%	13%
Pd@TiO_2_ (2% Pd)	49%	37%
Cu@TiO_2_ (0.9% Cu)	34%	19%
CuPd@TiO_2_ (0.8% Cu and 0.05% Pd)	37%	20%

**Table 3:** Conversion and selectivity for the alkynes in Scheme 2 over time for 8 mL of 6 mM solutions in EtOH stirred with 10 mg of Pd@TiO_2_ in Pyrex tubes and irradiated with 25% UVA power under Ar.

Item ↓/Molecule →	PhA	PhP	DPhA	DPhB
Time for 95% conversion, t_95_	67 min	60 min	28 min	114 min
Selectivity at 95% conversion	95%	88%	79%	48%
Selectivity at 1.5 × t_95_	52%	19%	11%	<1%
Selectivity at 2 × t_95_	23%	<0.5%	<0.2%	~0

**Table 4:** Comparison of catalyst performance under the conditions of Figure 8.

Catalyst	Alkyne Rate of Consumption (min^−1^)	Selectivity at 95% Alkyne Conversion	Selectivity at Twice the Time for 95% Conversion
Cu@TiO_2_	0.19	>99%	84%
CuPd@TiO_2_	0.29	>99%	97%
Pd@TiO_2_	3.94	88%	<1%

## Data Availability

Original data are available from the corresponding author upon reasonable request.

## Supplementary Materials

The following supporting information can be downloaded from https://www.mdpi.com/article/10.3390/nano13172390/s1: Figures S1–S13: GC-MS characterization, results for adsorption experiments of PhP at 900 ppm, spectral irradiance of the UVA source employed in the semi-hydrogenation reactions under different conditions, TEM images and the corresponding size histogram SEM/EDS analysis, and conversion of alkynes and selectivity toward alkenes at different conditions. Control experiments without light and with TiO_2_ itself have been added to show the effect of the used metals.
