# Supplementary material for: Photocatalytic Semi-Hydrogenation of Alkynes: A Game of Kinetics, Selectivity and Critical Timing
Source: Nanomaterials (Basel). 2023 Aug 22;13(17):2390. doi: 10.3390/nano13172390 (PMC10490202; doi:10.3390/nano13172390)
Supplement: Supplementary file 1 [file nanomaterials-13-02390-s001.zip › nanomaterials-2545301-supplementary.pdf]

Photocatalytic semihydrogenation of alkynes: a game of kinetics, selectivity and critical timing

Melissa Cely-Pinto, Bowen Wang and Juan C. Scaiano

Department of Chemistry and Biomolecular Sciences, University of Ottawa, Ottawa, ON K1N 6N5, Canada

Department of Chemistry and Biomolecular Sciences, University of Ottawa, Ottawa, ON K1N 6N5, Canada

Contents

Figure S1. Illustrating the effect of the relative values of k_1 and k_2 on the selectivity of a reaction. For panels A, B and C: The vertical blue dashed line corresponds to 99% conversion. Red line for alkyne, blue for alkene, green for alkane and black dash for % alkene selectivity. Panel D shows the selectivities against percent conversion, in red for $k_1 = 0.1\ k_2$, in green for $k_1 = 10\ k_2$ and in blue for $k_1 = 100\ k_2$. 4

Figure S2. Chromatograms showing the peaks of the alkynes used in this research. The alkynes have the following retention times: PhP (5.56 min), PhA (3.70 min), DPhA (9.86 min), DPhB (11.82 min)........ 4

Figure S3. Mass spectra of the alkynes. Each spectrum shows (   ) the molecular ion of the molecules. 5

Figure S4. Adsorption experiments of PhP using the catalysts used. **Conditions:** 10 mg Catalyst; 1 mL of 900 ppm alkyne solution in EtOH; **Stirring times:** 0 min, 10 min, 20 min, 30 min, 40 min. Aliquots were taken at selected times and filtered before using GC-MS to monitor the areas of PhP (5.56 min retention time identified by GC, Figure S1) at different times. Calibration curves for every alkyne were necessary to determine the concentration of the alkyne at the mentioned times using 3,5-di-tert-butyltoluene as an external standard. . 6

Figure S5. Irradiance of LED UVA source at different power of the used lamp. 7

Figure S6. Spectral Irradiance of UVA lamp used for semi-hydrogenation reactions; determined with a Luzchem spectroradiometer 7

Figure S7. Conversion of alkynes after performing hydrogenation reactions using alkyne solutions in EtOH at a concentration of 900 ppm, under argon and irradiated with UVA light at 25% power and with 10 mg Pd@TiO_2 suspended in 8 mL. Under these conditions, the order of relative consumption rates changes, with PhA showing the slowest consumption along DPhB while PhP is the fastest one. Note that when working in ppm, the concentration for all the alkynes ranges from 5.0 to 8.8 mM. 8

Figure S8. GC-chromatogram at 60 min of semi-hydrogenation reaction of PhP 6mM in EtOH using 10 mg of Pd@TiO_2 under Ar. At this point of time, 95% of the 1-phenyl-1-propyne (retention time of 5.56 min) has been consumed and *cis*-1-phenyl-1-propene (retention time of 4.90 min) is already formed. *Trans*-1-phenyl-1-propene (retention time of 5.22 min) is also formed but in trace amounts. For this chromatogram, a 0.1 mL aliquot of the reaction was taken. The solution was filtered to remove the catalyst and dissolved in 0.8 mL dichloromethane (DCM). Furthermore, 0.1 mL of a 2.4 mM solution of 3,5-Di-tert-butyltoluene (retention time of 7.7 min) was added to the vial as an external standard....... 8

Figure S9. Abundance of the alkynes (%Ky), its respective alkenes (%Ke) and alkane (%Ka) or selectivity towards the alkenes (%Sel int) vs. time. 8 mL of 6 mM solutions of the alkynes in EtOH were stirred with 10 mg of Pd@TiO_2 in Pyrex tubes and irradiated with 25% UVA power under Ar. **(A)** PhA, **(B)** PhP, **(C)** DPhA, **(D)** DPhB. 9

Figure S10. (A) Conversion of PhP after performing semi-hydrogenation reaction using a 6 mM alkyne solution in EtOH, under argon, irradiated with UVA light at 25% power, with 10 mg and 2 mg of Pd@TiO_2 suspended in 8 mL of solution; **(B)** Abundance of PhP (%Ky), its alkenes (%Ke) and alkane (%Ka) or selectivity of *cis*-1-phenyl-propyne (%Sel int) vs. time; 8 mL of a 6 mM solution in EtOH of PhP was stirred with 10 mg and 2mg of Pd@TiO_2 in different Pyrex® containers and irradiated with 25% UVA power under Ar. 9

Figure S11. (A) Abundance of PhP (%Ky), its respective alkene (%Ke) and alkane (%Ka) or selectivity towards the alkene (%Sel int) vs. time. 8 mL of 6 mM alkyne solution in MeOH was stirred with 10 mg of CuPd@TiO_2 and irradiated with 25% UVA power under Ar; **(B)** Abundance of PhP (%Ky), its respective alkene (%Ke) and alkane (%Ka) or selectivity towards the alkene (%Sel int) of PhP 6mM in MeOH using 5.4 mg of CuPd@TiO_2. 10

Figure S12. (A). Conversion of PhP after performing a control reaction using a 6 mM alkyne solution in MeOH, under argon, irradiated with UVA light at 100% power, with 10 mg of TiO_2 suspended in 8 mL of solution. At 540 min (9 h) of time, 4.6% of the 1-phenyl-1-propyne has been consumed. Aliquots were taken at different times to monitor the reaction through GC-MS.**(B)** GC-chromatograms at 0 min, 3 h and 9 h of the reaction using PhP 6 mM in MeOH and 10 mg of TiO_2 under Ar. At 540 min (9 h) the intensity of the peak for the 1-phenyl-1-propyne (peak at 5.56 min) has been decreased a little and the *cis*-1-phenyl-1-propene is barely formed (peak at of 4.90 min). For these chromatograms, a 0.1 mL aliquot of the reaction was taken. The solution was filtered to remove the catalyst and dissolved in 0.8 mL dichloromethane (DCM). Furthermore, 0.1 mL of a 2.4 mM solution of 3,5-di-tert-butyltoluene (retention time of 7.7 min) was added to the vial as an external standard..... 10

Figure S13. GC-chromatograms at 0 min, 3 h and 9 h of semi-hydrogenation control reaction of PhP 6 mM in MeOH using 10 mg of CuPd@TiO_2 under Ar without light. After 540 min (9 h), the 1-phenyl-1-propyne (retention time of 5.56 min) has not been consumed. Usually, when light is used as part of the reaction, at 9 h of reaction, all the alkyne was consumed and around 98% of *cis*-1-phenyl-1-propene (retention time of 4.90 min) was produced

(Figure S11A). The inset of the figure shows a zoom-in of the PhP peak to show that it remains the same after 9 h of reaction. For these chromatograms, a 0.1 mL aliquot of the reaction was taken. The solution was filtered to remove the catalyst and dissolved in 0.8 mL dichloromethane (DCM). Furthermore, 0.1 mL of a 2.4 mM solution of 3,5-di-tert-butyltoluene (retention time of 7.7 min) was added to the vial as an external standard..... 11

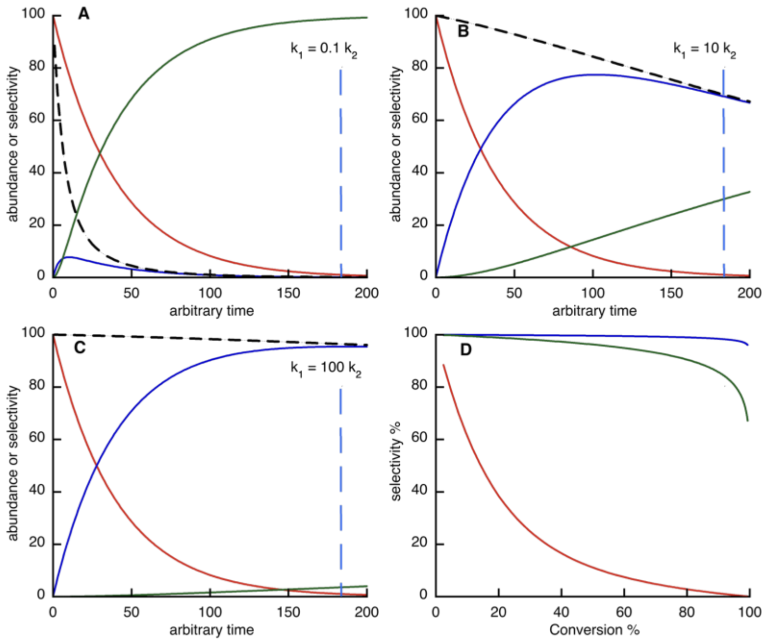

Figure S1. Illustrating the effect of the relative values of k_1 and k_2 on the selectivity of a reaction. For panels A, B and C: The vertical blue dashed line corresponds to 99% conversion. Red line for alkyne, blue for alkene, green for alkane and black dash for % alkene selectivity. Panel D shows the selectivities against percent conversion, in red for $k_1 = 0.1\ k_2$, in green for $k_1 = 10\ k_2$ and in blue for $k_1 = 100\ k_2$.

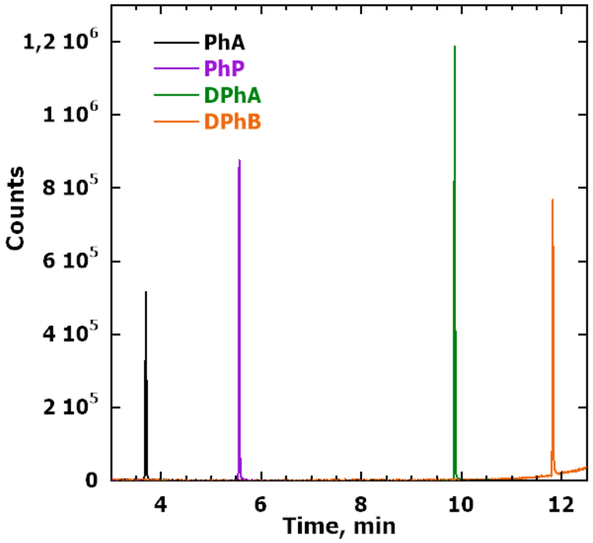

Figure S2. Chromatograms showing the peaks of the alkynes used in this research. The alkynes have the following retention times: PhP (5.56 min), PhA (3.70 min), DPhA (9.86 min), DPhB (11.82 min).

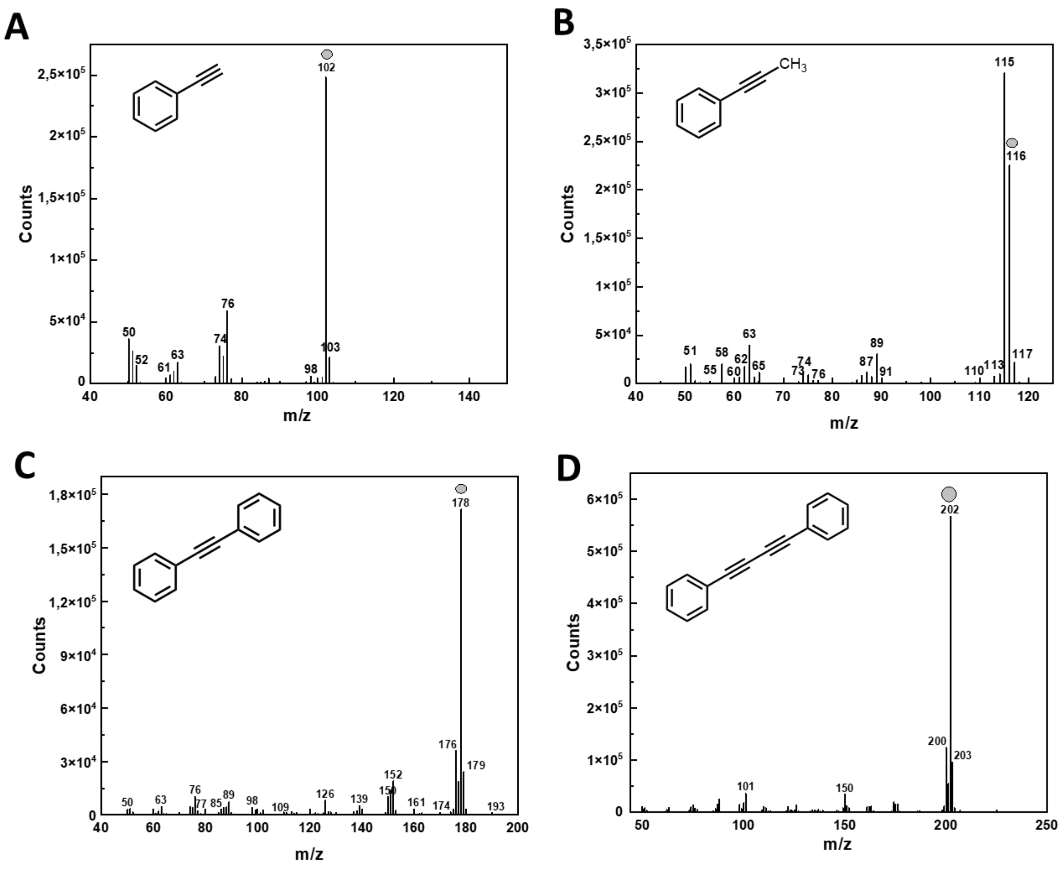

Figure S3. Mass spectra of the alkynes. Each spectrum shows (●) the molecular ion of the molecules.

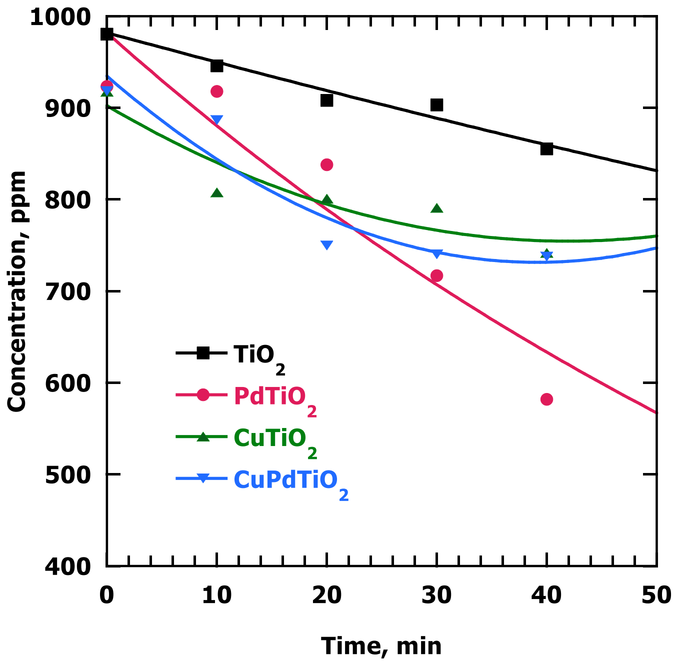

Figure S4. Adsorption experiments of PhP using the catalysts used. **Conditions:** 10 mg Catalyst; 1 mL of 900 ppm alkyne solution in EtOH; **Stirring times:** 0 min, 10 min, 20 min, 30 min, 40 min. Aliquots were taken at selected times and filtered before using GC-MS to monitor the areas of PhP (5.56 min retention time identified by GC, Figure S1) at different times. Calibration curves for every alkyne were necessary to determine the concentration of the alkyne at the mentioned times using 3,5-di-tert-butyltoluene as an external standard.

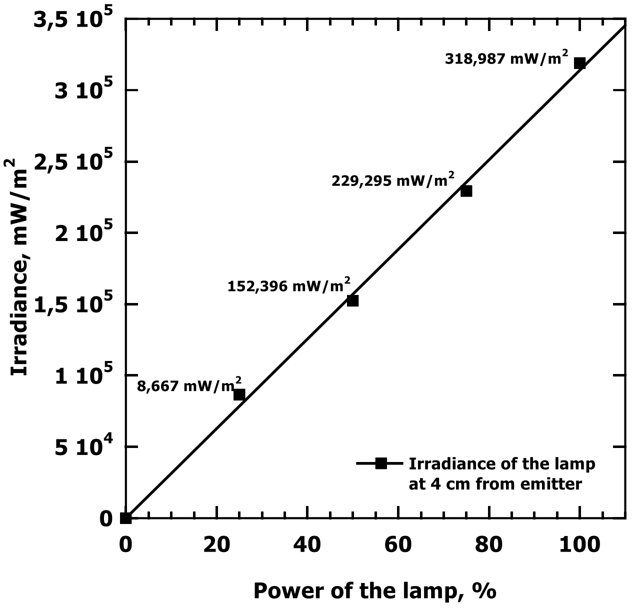

Figure S5. Irradiance of LED UVA source at different power of the used lamp.

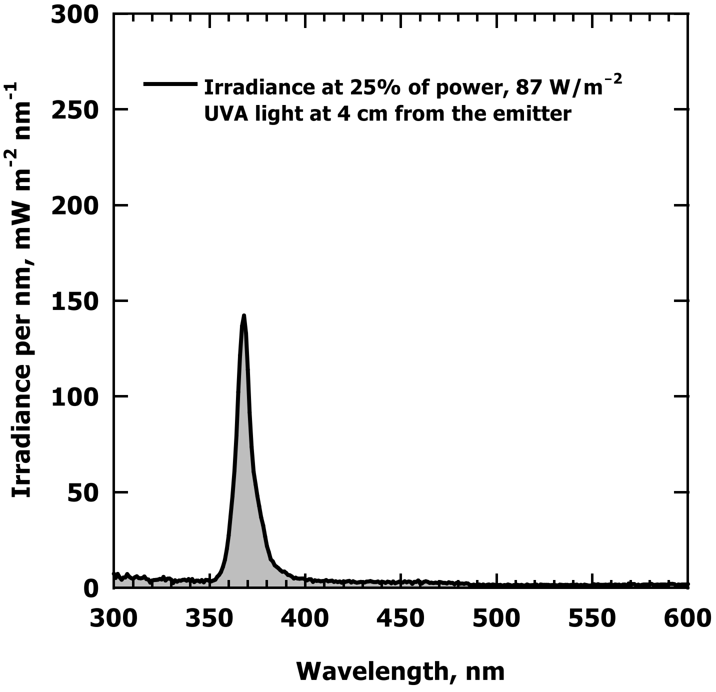

Figure S6. Spectral Irradiance of UVA lamp used for semi-hydrogenation reactions; determined with a Luzchem spectroradiometer

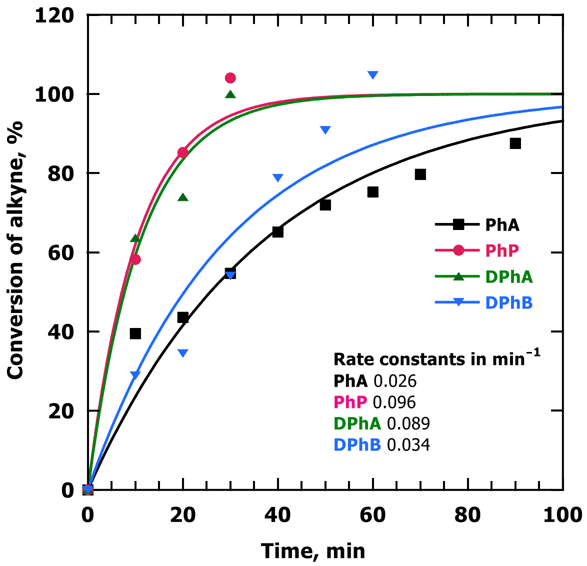

Figure S7. Conversion of alkynes after performing hydrogenation reactions using alkyne solutions in EtOH at a concentration of 900 ppm, under argon and irradiated with UVA light at 25% power and with 10 mg Pd@TiO_2 suspended in 8 mL. Under these conditions, the order of relative consumption rates changes, with PhA showing the slowest consumption along DPhB while PhP is the fastest one. Note that when working in ppm, the concentration for all the alkynes ranges from 5.0 to 8.8 mM.

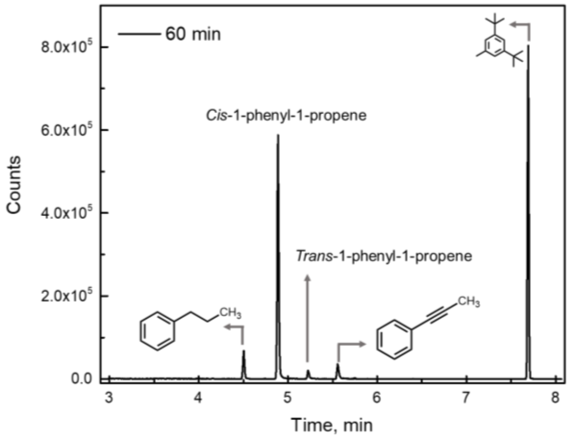

Figure S8. GC-chromatogram at 60 min of semi-hydrogenation reaction of PhP 6mM in EtOH using 10 mg of Pd@TiO_2 under Ar. At this point of time, 95% of the 1-phenyl-1-propyne (retention time of 5.56 min) has been consumed and *cis*-1-phenyl-1-propene (retention time of 4.90 min) is already formed. *Trans*-1-phenyl-1-propene (retention time of 5.22 min) is also formed but in trace amounts. For this chromatogram, a 0.1 mL aliquot of the reaction was taken. The solution was filtered to remove the catalyst and dissolved in 0.8 mL dichloromethane (DCM). Furthermore, 0.1 mL of a 2.4 mM solution of 3,5-Di-tert-butyltoluene (retention time of 7.7 min) was added to the vial as an external standard.

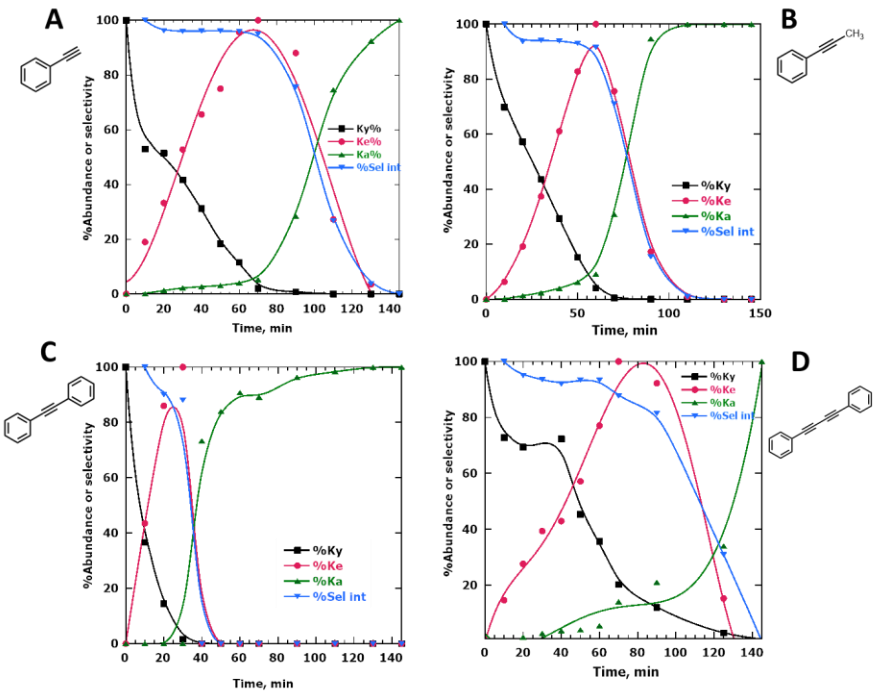

Figure S9. Abundance of the alkynes (%Ky), its respective alkenes (%Ke) and alkane (%Ka) or selectivity towards the alkenes (%Sel int) vs. time. 8 mL of 6 mM solutions of the alkynes in EtOH were stirred with 10 mg of Pd@TiO_2 in Pyrex tubes and irradiated with 25% UVA power under Ar. **(A)** PhA, **(B)** PhP, **(C)** DPhA, **(D)** DPhB.

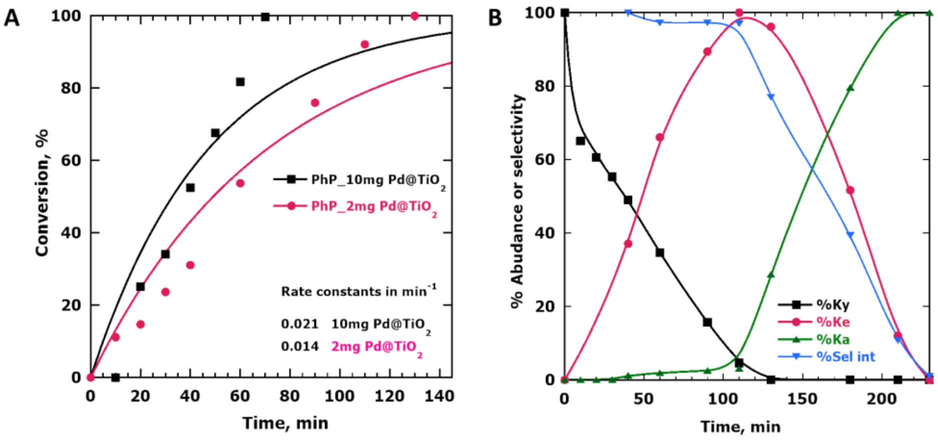

Figure S10. (A) Conversion of PhP after performing semi-hydrogenation reaction using a 6 mM alkyne solution in EtOH, under argon, irradiated with UVA light at 25% power, with 10 mg and 2 mg of Pd@TiO_2 suspended in 8 mL of solution; **(B)** Abundance of PhP (%Ky), its alkenes (%Ke) and alkane (%Ka) or selectivity of *cis*-1-phenyl-propyne (%Sel int) vs. time; 8 mL of a 6 mM solution in EtOH of PhP was stirred with 10 mg and 2mg of Pd@TiO_2 in different Pyrex® containers and irradiated with 25% UVA power under Ar.

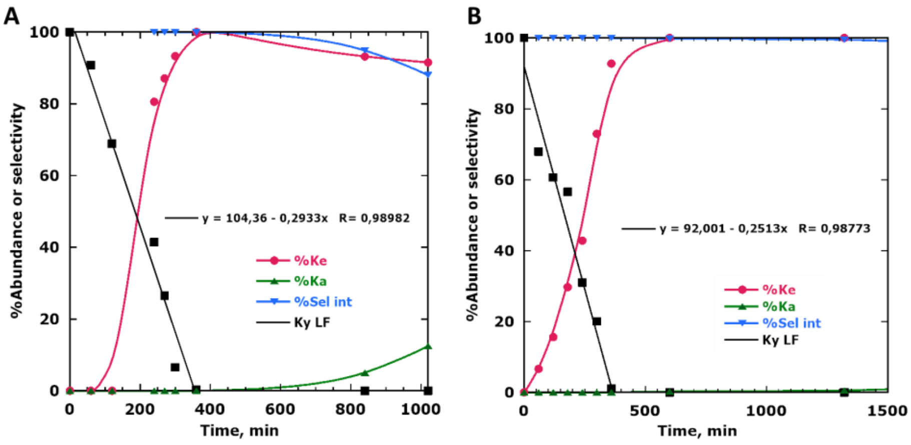

Figure S11. (A) Abundance of PhP (%Ky), its respective alkene (%Ke) and alkane (%Ka) or selectivity towards the alkene (%Sel int) vs. time. 8 mL of 6 mM alkyne solution in MeOH was stirred with 10 mg of CuPd@TiO_2 and irradiated with 25% UVA power under Ar; **(B)** Abundance of PhP (%Ky), its respective alkene (%Ke) and alkane (%Ka) or selectivity towards the alkene (%Sel int) of PhP 6mM in MeOH using 5.4 mg of CuPd@TiO_2.

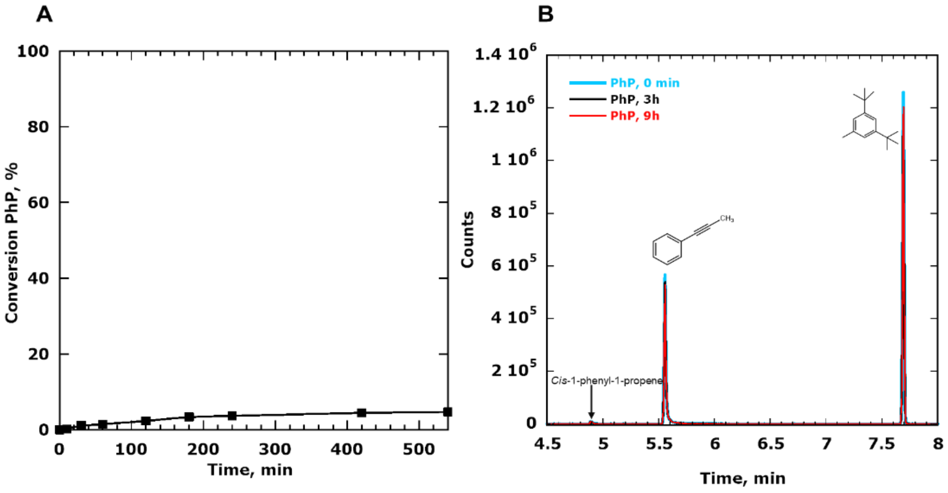

Figure S12. (A). Conversion of PhP after performing a control reaction using a 6 mM alkyne solution in MeOH, under argon, irradiated with UVA light at 100% power, with 10 mg of TiO_2 suspended in 8 mL of solution. At 540 min (9 h) of time, 4.6% of the 1-phenyl-1-propyne has been consumed. Aliquots were taken at different times to monitor the reaction through GC-MS.**(B)** GC-chromatograms at 0 min, 3 h and 9 h of the reaction using PhP 6 mM in MeOH and 10 mg of TiO_2 under Ar. At 540 min (9 h) the intensity of the peak for the 1-phenyl-1-propyne (peak at 5.56 min) has been decreased a little and the *cis*-1-phenyl-1-propene is barely formed (peak at of 4.90 min). For these chromatograms, a 0.1 mL aliquot of the reaction was taken. The solution was filtered to remove the catalyst and dissolved in 0.8 mL dichloromethane (DCM). Furthermore, 0.1 mL of a 2.4 mM solution of 3,5-di-tert-butyltoluene (retention time of 7.7 min) was added to the vial as an external standard.

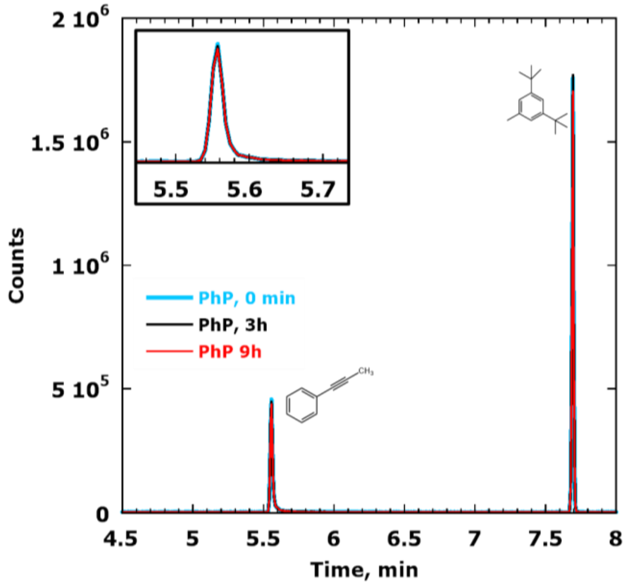

Figure S13. GC-chromatograms at 0 min, 3 h and 9 h of semi-hydrogenation control reaction of PhP 6 mM in MeOH using 10 mg of CuPd@TiO_2 under Ar without light. After 540 min (9 h), the 1-phenyl-1-propyne (retention time of 5.56 min) has not been consumed. Usually, when light is used as part of the reaction, at 9 h of reaction, all the alkyne was consumed and around 98% of *cis*-1-phenyl-1-propene (retention time of 4.90 min) was produced (Figure S11A). The inset of the figure shows a zoom-in of the PhP peak to show that it remains the same after 9 h of reaction. For these chromatograms, a 0.1 mL aliquot of the reaction was taken. The solution was filtered to remove the catalyst and dissolved in 0.8 mL dichloromethane (DCM). Furthermore, 0.1 mL of a 2.4 mM solution of 3,5-di-tert-butyltoluene (retention time of 7.7 min) was added to the vial as an external standard.
